# Microbial Diversity and Metabolite Profiles of Palm Wine Produced From Three Different Palm Tree Species in Côte d’Ivoire

**DOI:** 10.1038/s41598-020-58587-2

**Published:** 2020-02-03

**Authors:** Theodore N. Djeni, Karen H. Kouame, Francine D. M. Ake, Laurent S. T. Amoikon, Marcellin K. Dje, Kumaraswamy Jeyaram

**Affiliations:** 1 Laboratoire de Biotechnologie et Microbiologie des Aliments, Unité de Formation et de Recherche en Sciences et Technologie des Aliments (UFR-STA), Université Nangui Abrogoua, 02 BP 801 Abidjan 02, Abidjan, Côte d’Ivoire; 2 Microbial Resources Division, Institute of Bioresources and Sustainable Development (IBSD), Takyelpat Institutional Area, Imphal, 795 001 Manipur India

**Keywords:** Biotechnology, Microbiology

## Abstract

Palm wine, the most commonly consumed traditional alcoholic beverage in Western Africa, harbours a complex microbiota and metabolites, which plays a crucial role in the overall quality and value of the product. In the present study, a combined metagenomic and metabolomic approach was applied to describe the microbial community structure and metabolites profile of fermented saps from three palm species (*Elaeis guineensis*, *Raphia hookeri*, *Borassus aethiopum*) in Côte d’Ivoire. *Lactobacillaceae* (47%), *Leuconostocaceae* (16%) and *Acetobacteriaceae* (28%) were the most abundant bacteria and *Saccharomyces cerevisiae* (87%) the predominant yeasts in these beverages. The microbial community structure of *Raphia* wine was distinctly different from the others. Multivariate analysis based on the metabolites profile clearly separated the three palm wine types. The main differentiating metabolites were putatively identified as gevotroline hydrochloride, sesartemin and methylisocitrate in *Elaeis* wine; derivative of homoserine, mitoxantrone in *Raphia* wine; pyrimidine nucleotide sugars (UDP-D-galacturonate) and myo-Inositol derivatives in *Borassus* wine. The enriched presence of gevotroline (an antipsychotic agent) and mitoxantrone (an anticancer drug) in palm wine supports its therapeutic potential. This work provides a valuable insight into the microbiology and biochemistry of palm wines and a rationale for selecting functional microorganisms for potential biotechnology applications.

## Introduction

Palm wine is a collective name for a group of alcoholic beverages produced from the palm tree sap by natural fermentation. Various species of Palmae family^1^, such as oil palm (*Elaeis guineensis*), raffia palm (*Raphia hookeri*), date palm (*Phoenix dactylifera*), ron palm (*Borassus aethiopum*) and coconut palm (*Cocos nucifera*)^2–6^ are generally used for the palm wine production in Africa. Palm wine is the most commonly consumed traditional alcoholic beverage in Western Africa^7^, with an estimate of more than 10 million people consuming it^8,9^. Moreover, palm wine tapping is an important commercial activity for rural livelihood in the region^10,11^. As part of cultural heritage, palm wine is especially used in traditional namings and marriage ceremonies, traditional incantations and in folk medicines. Interestingly, both government and health professionals have recognized the importance of palm wine in the treatment of malnutrition in the region. Palm wine is rich in trace elements and vitamins, traditionally believed for the general wellbeing of the lactating women^12^. Moreover, palm wine is well known for its anti-oxidant properties and its health-promoting benefits are demonstrated in rat models^13^. Consequently, the palm wine production and trade represents an important source of income in West Africa, as it employs nearly three-quarters of the male population in some villages, producing about 10,000 L per day and providing a monthly per capita income of about $ US 40–70^14^.

**Figure 1:**
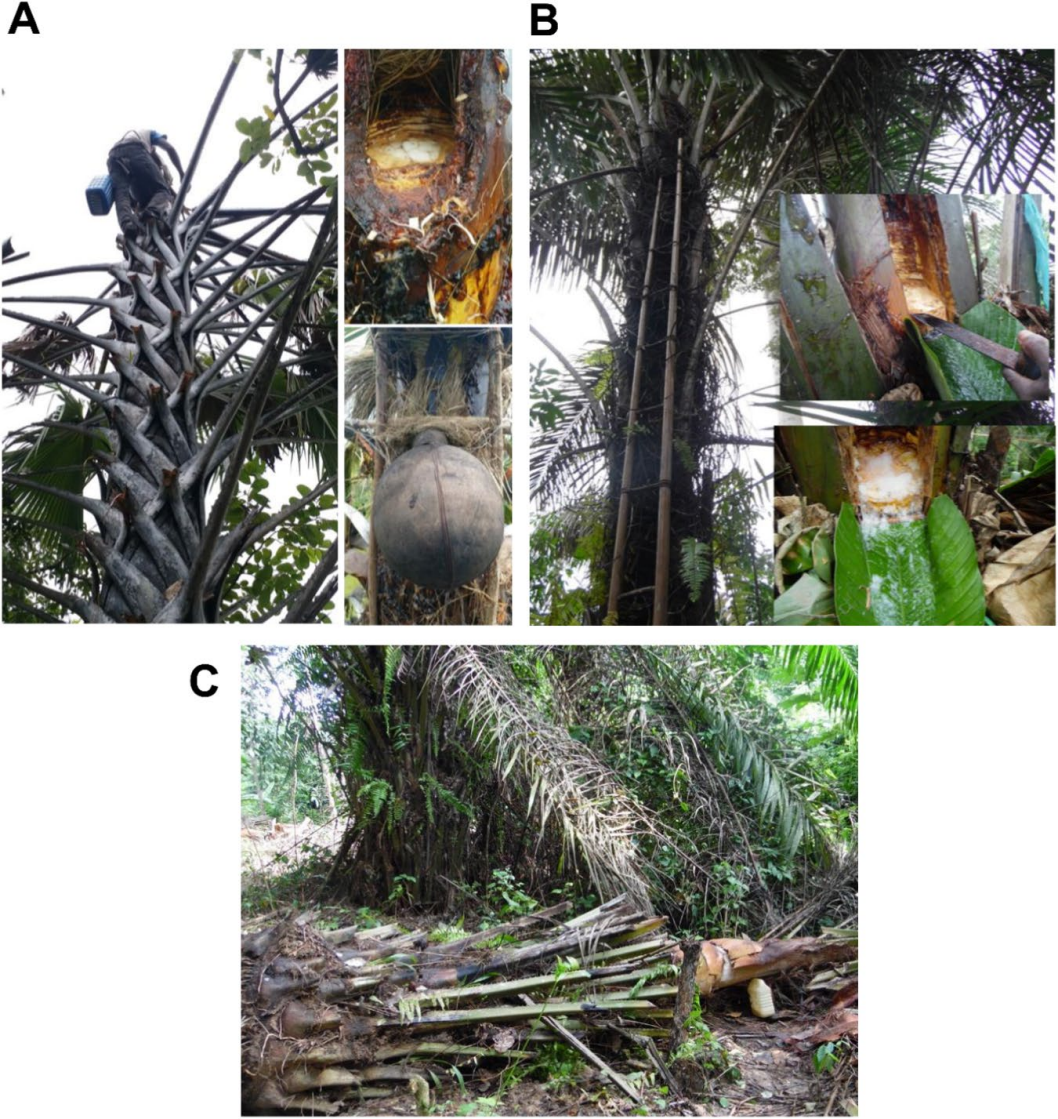
The indigenous process of destructive palm sap tapping practice in the savanna area of Côte d’Ivoire for palm wine production from ron palm (*Borassus aethiopum*) **(A)**, raffia palm (*Raphia hookeri*) **(B)** and oil palm (*Elaeis guineensis*) **(C)**. Perforation of the apical meristem of the tree trunk is in practice for sap tapping from ron palm and raffia palm, whereas the oil palm tree is uprooted before tapping.

Traditionally, non-destructive methods (palm wine tapping from the palm tree inflorescence) and destructive methods (incision on apical meristem of palm tree or trunk of the felled palm tree) are in practice for the palm sap collection in West Africa^15,16^. The destructive palm wine tapping process involves perforation or cavity digging into the soft apical meristem of the tree trunk and insertion of a tube or making way for sap collection in a container (traditionally in a calabash pot or terracotta clay pot and recent days in plastic containers). The indigenous processes of destructive palm wine tapping from ron palm (*Borassus aethiopum*), raffia palm (*Raphia hookeri*) and oil palm (*Elaeis guineensis*) in the savanna and forest areas of Côte d’Ivoire are shown in Fig. 1. In case of oil palm wine tapping, the tree is uprooted before tapping. During this process, the tree will die and heavy destruction of palm tree population due to excessive palm wine tapping is reported in the region^17^. The palm sap is a nutrient-rich medium that harbours a complex microbiota which includes the native resident flora and the invader flora brought by insects, palm sap collection containers and tappers. The native flora is mostly influenced by the palm tree species and its geographical location^18^. This diverse range of yeast and bacterial communities^4,5^ ferment the sugars present in the palm sap^19^. During the process, they produce a wide variety of secondary metabolites thereby influence the sensory properties and thus create diversification of palm wine with different taste and aroma^20^.

In previous studies, several microorganisms have been isolated and characterised from palm wines^4,5,16^. These conventional microbiology methods have provided a valuable but rather incomplete picture on the microbial ecology of palm wine fermentation. Therefore, the application of culture-independent metagenomic approaches may provide a more comprehensive view of the microbial communities associated with palm wines^21,22^. Next-Generation Sequencing (NGS) techniques based ribosomal rRNA gene amplicon sequencing has been widely applied and resulted in a new insight into the microbial community of naturally fermented alcoholic beverages including grape and botrytized wine^23,24^ and rice wine^25,26^. However, the culture-independent method has been rarely used for studying the microbial ecology of palm wine fermentation. Two recent NGS-based studies reported the bacterial diversity in palm wine of Nigeria^27^ and coconut palm wine of Mexico^28^. However, NGS based comparative analysis of palm wine yeast diversity along with bacterial diversity is not reported. Several researchers compared the volatile compounds of palm wine produced from different palm tree species by GC-MS analysis^29,30^. Also, very few reports are available about the changes in the metabolites during palm sap fermentation to palm wine analysed by LC-MS^31^. In this study, we used culture-independent molecular approach of MiSeq amplicon sequencing of ribosomal rRNA gene and fungal ITS region to compare the bacterial and yeast community structure together and LC-HRMS analysis to compare the metabolites profile of wine produced from three different palm tree species in Côte d’Ivoire, by taking into consideration the diversity of production sources. We aimed to identify the key microbial communities and metabolites associated with the palm wines produced from ron palm (*Borassus aethiopum*), oil palm (*Elaeis guineensis*) and raffia palm (*Raphia hookeri*) in Côte d’Ivoire and attempted to establish the basis of their differences.

## Results

### Microbial community structure of palm wines

**Figure 2:**
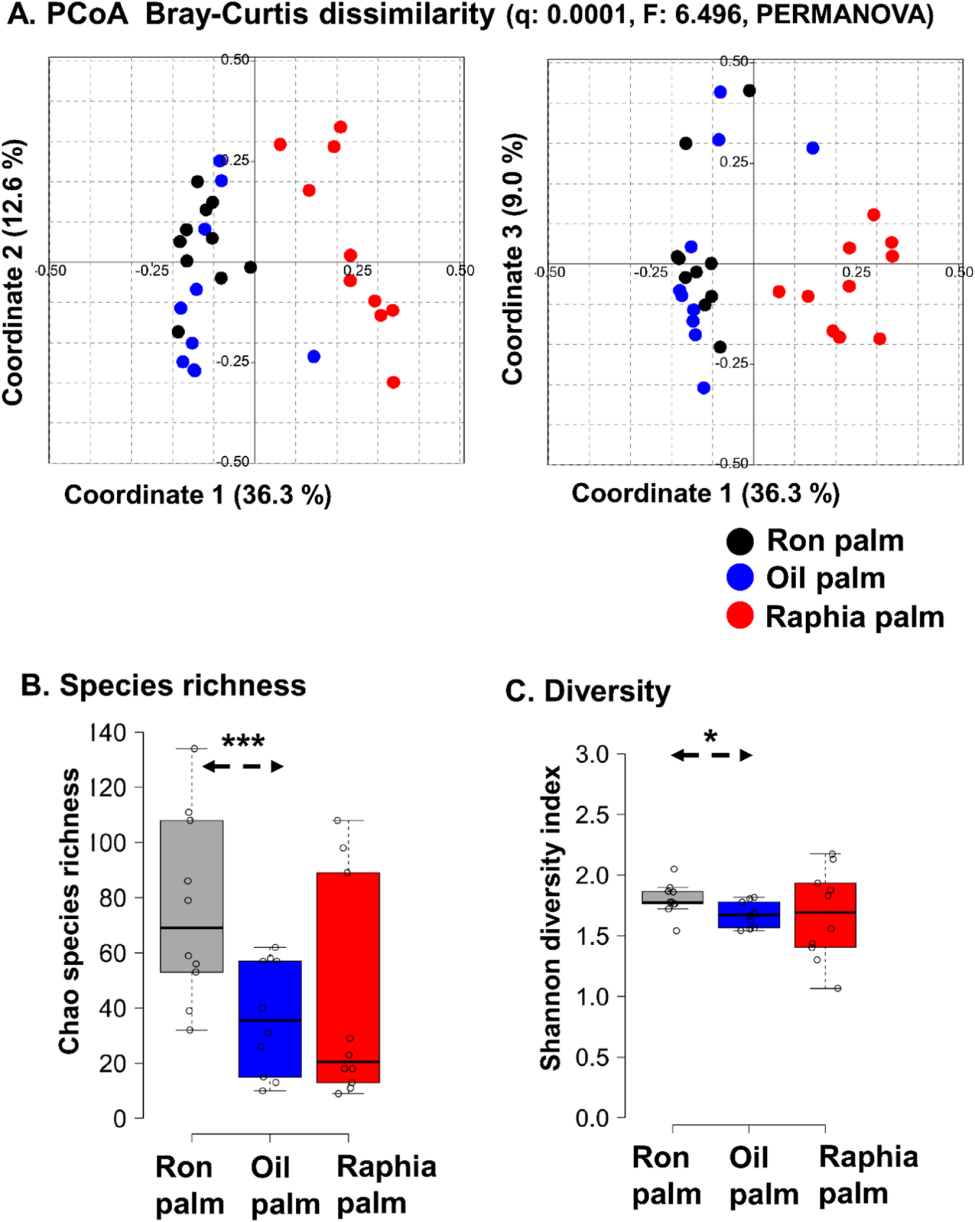
The difference in the overall microbial community structure and diversity present in palm wines produced from three palm tree species. (**A**) PCoA using Bray–Curtis dissimilarity based on the species-level OTUs shows a significant difference between the palm wine samples based on the tree species. The significance in the difference expressed as Bonferroni corrected p-values (q = 0.0001, F = 6.5, PERMANOVA; q = 0.0001, R = 0.4335, ANOSIM). PERMANOVA: Permutational multivariate analysis of variance; ANOSIM: Analysis of similarities. (**B**) The boxplot shows the existence of high microbial diversity in the ron palm wine (Chao species richness and Shannon diversity) than the other two palm wine types. The significance in the difference was calculated by Students t-test, 2 tailed (indicated as *p < 0.05, ***p < 0.001).

The bacterial and yeast communities of naturally fermented saps collected from three palm tree species (*Elaeis guineensis*, *Raphia hookeri*, and *Borassus aethiopum*) during palm wine production in Côte d’Ivoire were analysed for the first time by MiSeq amplicon sequencing of the bacterial V3-V4 region of 16S rRNA gene and fungal ITS region. This in-depth analysis resulted in a total of 1,981,699 quality-filtered sequences of 450 bp read length with an average read of 16,989 ± 9,260 per sample for bacteria and 1,851,521 quality-filtered sequences of 200–450 bp read length with an average read of 23,037 ± 7,046 per sample for yeasts. The yeast sequence distribution showed 80.6% sequences with a read length of 476–479 bp and 16.2% sequences with a read length of 399–409 bp. We compared the overall microbial community structure (bacteria and yeast) present in the three types of palm wine samples (n = 10 for each palm species) by PCoA using Bray–Curtis dissimilarity based on the species-level OTUs relative abundance profiles. The microbial community structure in these palm wine samples significantly differed (q = 0.0001, F = 6.5, PERMANOVA; q = 0.0001, R = 0.4335, ANOSIM) (Fig. 2A). The results showed a distinct separation in the microbial community structure of raphia wine from the oil palm and ron palm wine samples (q = 0.0006, Pair-wise ANOVA, Bonferroni corrected). However, the alpha diversity analysis (Chao species richness and Shannon diversity index) showed a high microbial diversity in the ron wine samples (Fig. 2B,C) than the oil palm wine (p < 0.05, Students t-test, two-tailed), and the low diversity noticed in the raphia wine was statistically not significant.

**Figure 3:**
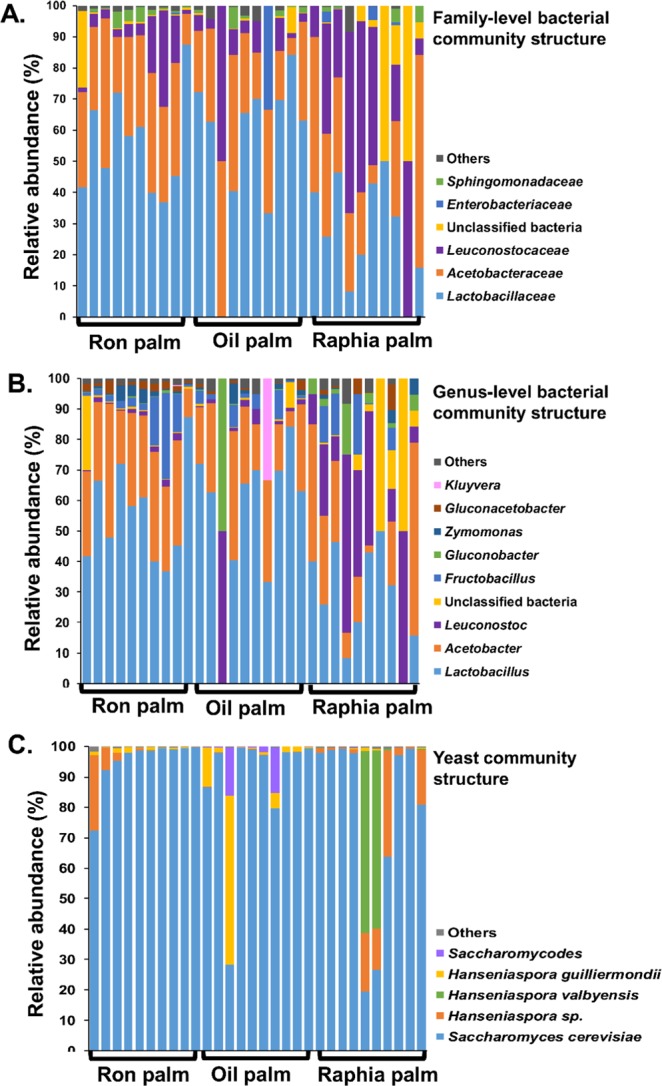
The taxon bar chart shows the difference in the relative abundance (%) of predominant bacteria at family-level (**A**), bacteria at genus-level (**B**) and yeast species (**C**) present in the three types of palm wine. The taxa with a mean relative abundance of less than 1% across the samples are combined and shown as others.

The bacteria and yeasts compositional difference between the three palm wines types was also visualised by comparing the relative abundance of the predominant taxa at different taxonomic levels (Fig. 3A–C). The predominant bacterial phyla present in the three types of palm wine were Firmicutes (68.8%) and Proteobacteria (32.6%). The Firmicutes phylum was represented mainly by *Lactobacillaceae* (46.6%) and *Leuconostocaceae* (15.9%), while the Proteobacteria phylum included *Acetobacteraceae* (27.8%), *Sphingomonadaceae* (1.4%) and *Enterobacteriaceae* (1.4%) (Fig. 3A). A substantial part (5.9%) of unclassified bacteria was also present in the palm wines. Further analysis showed *Lactobacilliaceae* as the key differentiating taxa (q < 0.001, Wilcoxon test, BH corrected), with a high presence (56% relative abundance) in ron palm and oil palm wines than raphia palm wine (28% relative abundance). At the genus level, *Lactobacillus* (46.6%), *Acetobacter* (23.4%), *Leuconostoc* (10.6%), *Fructobacillus* (5.2%), *Gluconobacter* (3.6%), *Zymomonas* (1.4%), *Gluconoacetobacter* (1.4%) and *Kluyvera* (1.1%) were the relatively abundant genera in the palm wine samples (Fig. 3B). A high presence of *Leuconostoc* and low presence of *Lactobacillus* (q < 0.01, Wilcoxon test, BH corrected) in raphia palm wine than the other two palm wines appeared as the key difference.

**Figure 4:**
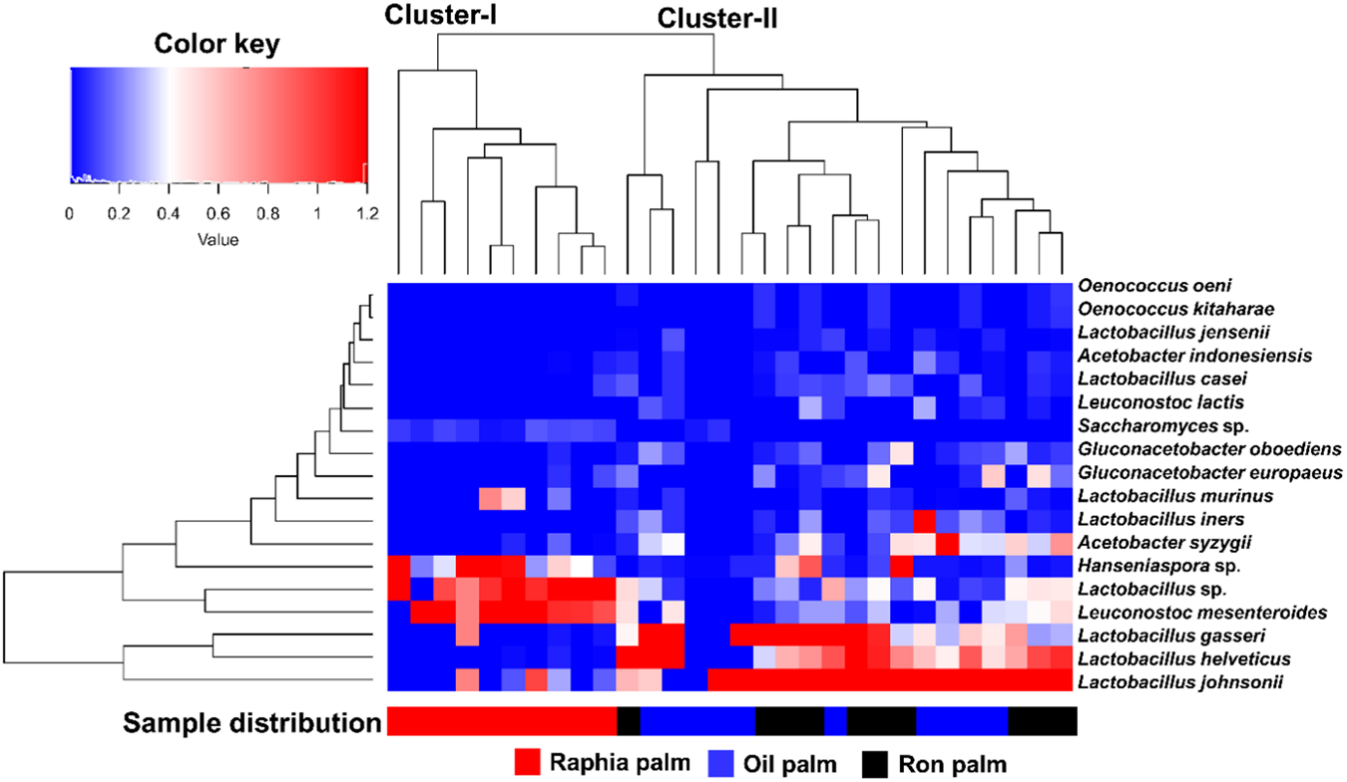
A hierarchically clustered heat map shows the microbial species-level differential abundance in three palm wine types. The significantly differencing 18 bacterial and yeast species (q < 0.0001, Wilcoxon test, BH corrected) between the wine types are clustered here. The abundance difference is shown as a color key with a red and blue colour gradient. The sample distribution of different types of wines over the clusters (Clusters I and II) is shown below the heat map.

The predominant yeast taxa associated with these palm wine samples are shown with a relative abundance graph at the species level (Fig. 3C). *Saccharomyces cerevisiae* dominated in all three types of palm wine with a relative abundance of 95.3%, 88.4% and 78.1% in ron palm, oil palm and raphia palm wines respectively. *Hanseniaspora* sp. (9.28%) and *Hanseniaspora valbyensis* (11.92%) were the next two predominant yeast species present in raphia palm wine, while *Hanseniaspora guilliermondii* (8.0%) and *Saccharomycodes* (3.4%) were present in oil palm wine. Pairwise comparison showed a higher abundance of *Hanseniaspora* in raphia palm wine (q = 0.02, Wilcoxon test, BH corrected). A clustered heat map based on the relative abundance of significantly differing predominant bacterial and yeast species between three types of palm wines is shown in Fig. 4. As shown in this figure, the palm wine samples analyzed were divided into two clusters (Cluster-I and Cluster-II). The Cluster-I composed of raphia palm wine samples, characterized by the presence of *Leuconostoc mesenteroides*, *Lactobacillus* sp., *Hanseniaspora* sp., and *Saccharomyces*. The Cluster-II, constituted by samples of both oil palm and ron palm wines, predominated by *Lactobacillus johnsonii*, *Lactobacillus helveticus*, *Lactobacillus gasseri*, *Acetobacter syzygii*, *Lacctobacillus iners*, and *Gluconacetobacter*. Above analysis showed a distinct microbial community profile in raphia palm wine than other two palm wines studied.

### Metabolite profile of palm wines

**Figure 5:**
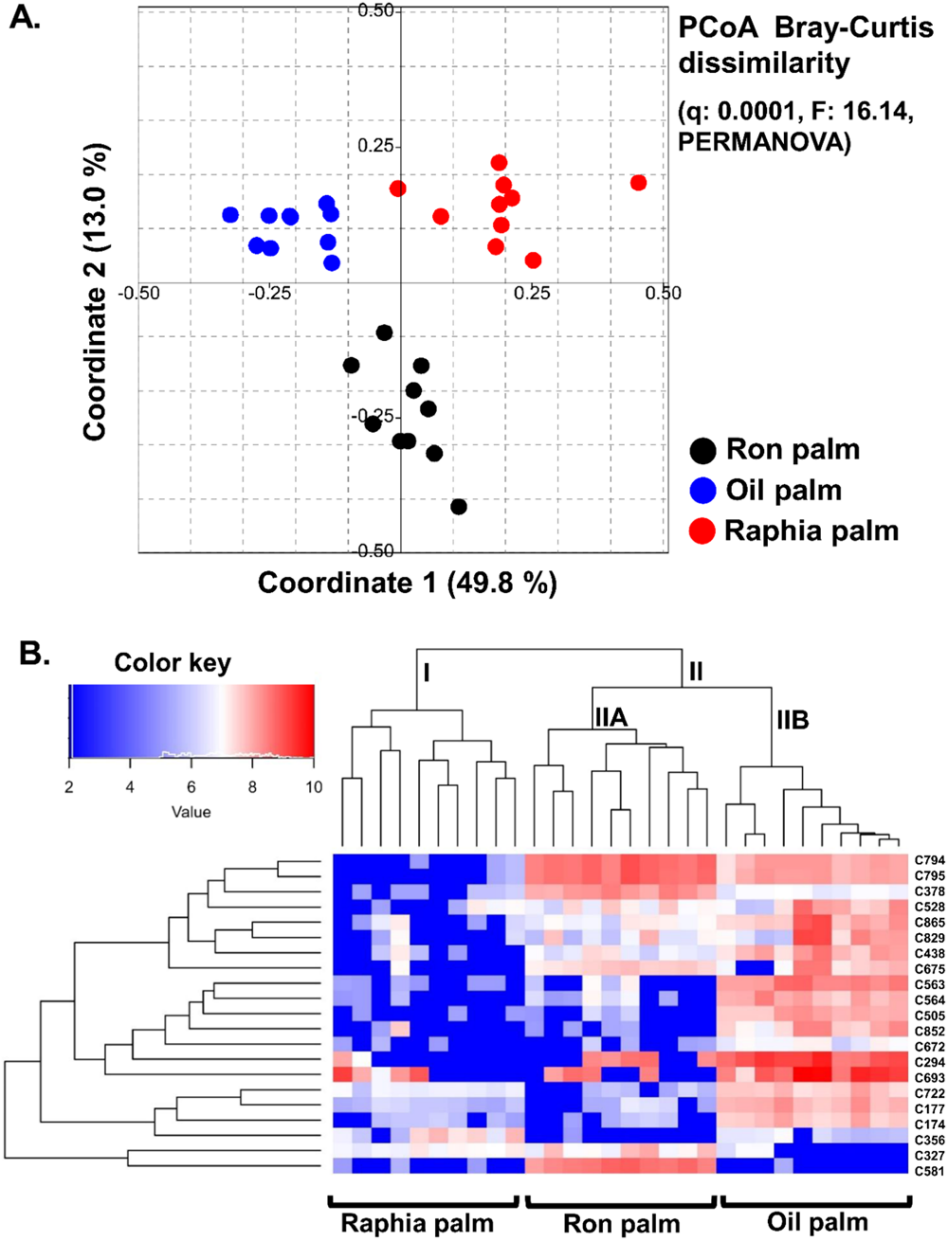
Comparison of the metabolite profiles of three different types of palm wines. (**A**) PCoA using Bray–Curtis dissimilarity based on the palm wine metabolites profile generated by LC-HRMS shows a significant difference between the palm wine samples based on the palm tree species. The significance in the difference expressed as Bonferroni corrected p-values (q: 0.0001, F: 16.14, PERMANOVA). (**B**) The hierarchically clustered heat map shows the clustering of three different palm wine types based on the significantly differencing 21 metabolites (log2 fold change of >1 and q < 0.0001, Wilcoxon test, BH corrected). The concentration difference in the metabolites is shown with a red and blue colour gradient.

We compared the metabolites present in the palm wines produced from the three different palm tree species by liquid chromatography-high resolution mass spectrometry (LC-HRMS) analysis. A total of 765 metabolites obtained after quality filtration of the data was assigned the identity of the possible compounds by Human Metabolome DataBase/Kyoto Encyclopedia of Genes and Genomes (HMDB/KEGG) database. Further, based on the quality control (QC) of pooled samples coefficient of variation (CV), the data with CV_QC > 20% were removed and limited to 338 components. The metabolite profiling showed that the palm wines were enriched with organic acids (lactic acid), hexose deoxy sugars (fucose), sugar alcohols (sorbitol) and sugar acids (gluconic acid). PCoA using Bray-Curtis dissimilarity analysis based on the overall metabolite profile was used to compare the three palm wines types. The results showed a clear separation (q = 0.0001, F = 16.14, PERMANOVA) among the three palm wine types along with coordinate-1 which accounted for 49.8% of the total variations **(**Fig. 5A**)**. Further, the pair-wise Wilcoxon test resulted in 21 metabolites that were significantly different between the three palm wine types (Table 1**)**. The hierarchical clustering analysis performed to visualise the significantly differentiating metabolites (Fig. 5B) formed two main clusters (I and II). Similar to the microbial community analysis, the Cluster-I constitute the samples of raphia palm wine which was distinctly different from the other two wine types. The Cluster-II further segregated into ron palm wine (IIA) and oil palm wine (IIB) samples. Our analysis did not find the exact mass match with 4 decimal accuracy and MS/MS spectral profile with the available chemical database for many of the palm wine metabolites. From the abundancy based on the peak area, the main differentiating metabolites with putative identification were methylisocitrate, sesartemin, gevotroline hydrochloride in oil palm wine; derivative of homoserine, mitoxantrone in raphia palm wine; pyrimidine nucleotide sugars and myo-Inositol derivatives in ron palm wine. Among the identified compounds, the higher relative concentration of gevotroline (an antipsychotic agent and a dopamine receptor D2 antagonist) and mitoxantrone (an anticancer drug) present in the palm wine supported its therapeutic potential. The closest matches for the key differentiating metabolites exclusively present in the ron palm wine samples were the derivatives of pyrimidine nucleotide sugars (UDP-D-galacturonate, UDP-D-xylose, and UDP-L-arabinose). The detection of ammonium persulphate in the oil palm and raphia palm wines (not in the ron palm wine) might be linked with the post-production environmental contamination.

**Table 1:** Significantly differing metabolites between three palm wines analysed by LC-HRMS.

ID	RT (min)	MW	Closest Chemspider hits (within 20ppm)	Average relative abundance of palm wine metabolites (LC-HRMS data log-transformed)			Wilcoxon test, BH corrected p-values		
				Ron palm wine (R)	Oil palm wine (P)	Raphia palm wine (H)	R v P	R v H	P vs H
C174	4.35	152.0826	Isopropyl catechol;	2.95	7.67	4.07	0.0001	NS*	0.0006
			2-Isopropoxyphenol;						
			2,6,6-Trimethyl-2-cyclohexene-1,4-dione						
C177	4.35	154.0983	Methyl 2-octynoate;	3.59	7.91	5.79	0.0003	NS	0.0000
			Octahydrochromen-2-one;						
			2,2,6-Trimethyl-1,4-cyclohexanedione						
C294	6.63	206.0423	3-hydroxy-1,2,3-Butanetricarboxylic acid;	4.29	9.02	1.58	0.0032	NS	0.0000
			3-C-Carboxy-2,4-dideoxy-2-methylpentaric acid;						
			3-(Carboxymethyl)-3-hydroxypentanedioic acid						
C327	4.12	219.0739	O-(3-Carboxypropanoyl) homoserine;	7.26	2.17	6.95	0.0001	NS	0.0002
			O-Succinyl-L-homoserine;						
			N-[(3 S,4 R,5 S,6 R)-4,5-Dihydroxy-6-(hydroxymethyl)-2-oxotetrahydro-2H-pyran-3-yl]acetamide						
C356	2.83	227.9759	Ammonium persulfate	0.68	5.62	6.28	0.0000	0.0000	NS
C378	6.03	242.0190	1D-myo-Inositol 1,2-cyclic phosphate;	8.18	6.81	3.37	0.0000	0.0000	0.0013
			1-Deoxy-6-O-phosphono-D-threo-hexo-2,5-diulose						
C438	14.74	275.1121	gamma-Glu-gln;	6.73	7.90	2.46	0.0000	0.0004	0.0000
			Norophthalmic acid;						
			N(2)-Succinyl-L-citrulline						
C505	2.81	310.1818	Botrydial;	2.36	7.91	1.60	0.0000	NS	0.0000
			6-Hydroxy-4,9a-dimethoxy-3,4a,5-trimethyl-4a,5,6,7,8,8a,9,9a-octahydronaphtho[2,3-b]furan-2(4 H)-one;						
			4-(Acetoxymethyl)-7-methyl-1,4a,5,6,7,7a-hexahydrocyclopenta[c]pyran-1-yl 3-methylbutanoate						
C528	8.31	327.1182	Zolamine hydrochloride	7.03	7.45	3.23	NS	0.0024	0.0013
C563	12.17	345.1427	Gevotroline hydrochloride	2.66	8.40	3.14	0.0000	NS	0.0000
C564	12.18	347.1583	Methyl 4-(2-benzylbenzoyl)-2,5-dimethyl-1H-pyrrole-3-carboxylate	4.27	7.96	1.64	0.0009	NS	0.0000
C581	17.56	356.1323	4-O-Methylgalactinol;	8.56	0.71	1.64	0.0000	0.0000	NS
			(1 S,2 S,3 R,4 S,5 R,6 S)-2,3,4,6-Tetrahydroxy-5-methoxycyclohexyl beta-D-galactopyranoside						
C672	10.97	414.1742	9-Deoxyhymatoxin A	2.28	6.83	1.66	0.0001	NS	0.0000
C675	15.72	416.1536	Nelezaprine maleate	7.52	6.18	0.79	NS	0.0000	0.0004
C693	10.07	430.1689	Sesartemin;	3.50	9.01	4.17	0.0008	NS	0.0027
			(1 R,2 S,4 S,10 R,12 R,14 R,15 R)-7-Formyl-4-isopropenyl-12,14-dimethyl-17-oxo-11,16,18,19-tetraoxapentacyclo [12.2.2.1~6,9~.0~1,15~.0~10,12~]nonadeca-6,8-dien-2-yl acetate;						
			4-Methoxy-6-[4-(3,4,5-trimethoxyphenyl)tetrahydro-1H,3H-furo[3,4-c]furan-1-yl]-1,3-benzodioxole.						
C722	6.33	444.2000	Mitoxantrone	3.47	7.65	6.64	0.0003	0.0032	0.0000
C794	8.16	536.0452	UDP-D-xylose;	8.68	7.99	1.68	0.0000	0.0000	0.0000
			UDP-L-arabinose;						
			5”-O-[{[{[(2 R,3 R,4 R)-3,4-Dihydroxy-4-(hydroxymethyl) tetrahydro-2-furanyl]oxy}(hydroxy)phosphoryl]oxy} (hydroxy)phosphoryl]uridine						
C795	9.20	536.0451	UDP-D-galacturonate	8.67	7.98	1.18	0.0000	0.0000	0.0000
C829	15.01	592.2224	Unidentified	6.62	7.55	1.93	NS	0.0002	0.0000
C852	13.96	649.2013	UNII:73033I28Y6	2.20	7.71	1.35	0.0000	NS	0.0000
C865	23.51	726.2445	Unidentified	6.88	8.01	3.08	0.0001	0.0016	0.0002

^*^NS- denotes no significant difference.

## Discussion

Palm wine is a traditionally fermented drink, appreciated and consumed by several people throughout the world^32^, and which is suggested to contribute to the digestive wellbeing and proposed as folklore medicine^33^. As it is consumed with live fermenting microbiota, it clearly appears the importance to master its microbiota and chemical composition on a biotechnological point of view in one hand, and to ensure the safety and quality in another hand. Thus, NGS and LC-MS analyses were performed here to improve our understanding of the microbial community structure and metabolite profiles of palm wines produced from three different palm species. Moreover, this comparative analysis will highlight the distinctiveness of the palm wine produced from different palm tree species. Culture-independent NGS is a cost-effective approach to study the composition and dynamics of microbial communities during natural food fermentation^34,35^. However, to our knowledge, as of today only two studies have been published that employed NGS to study the bacterial diversity in the palm wine ecosystem^27,28^. Our study has substantially improved the understanding of the bacterial and yeast diversity present in the fermented sap from three palm tree species, mainly tapped for palm wine production in Côte d’Ivoire. The estimated high species richness (504 bacteria and 96 yeasts) and diversity (Shannon index 1.6 to 1.8) of microbiota in these palm wines surpass many of the previous culture-based studies^4,5^. The high throughput NGS gave a more comprehensive view at the microbial community of palm wines. The composition of palm wine can differ significantly as a result of differences in the tapping process^19^, palm tree species^12,36^ and the production area^37^. Our data showed a major variance in the microbial and metabolite profile of the raphia palm wine than the other two palm wine types despite the similar functional fermentation of sugary sap to palm wine. Also, noticed a high diversity of the fermenting microbiota in the ron palm wine samples collected from Toumodi (Centre region of Côte d’Ivoire) than the raphia palm and oil palm wines collected from Adzope (South Eastern Côte d’Ivoire).

Among the bacterial phylum, the dominance of Firmicutes in fermented foods and beverages is consistent with previous reports^26,38,39^. At the genus level, *Lactobacillus* and *Leuconostoc* account nearly 60% of the overall relative abundance in most of the palm wine samples studied from Côte d’Ivoire. As reported in several culture-based studies, *Lactobacillus* dominated the palm wine samples^4,5,16^. *Lactobacillus johnsonii* and *Lactobacillus helveticus* which were well known for their probiotic potential^40^ were found with a high abundance in ron palm and oil palm wines. Whereas, *Leuconostoc mesenteroides* was abundantly present in the raphia wine samples. Moreover, *Fructobacillus* dominantly present in the “tuba”- coconut palm wine of Mexico^27^ was present at low abundance (~5% relative abundance) in the palm wines of Côte d’Ivoire. Our results support the earlier findings of different dominant lactic acid bacteria (LAB) species according to the palm tree species studied^4,6,41^. Lactic acid bacteria are responsible for the pH reduction through production of organic acids, which give a sour taste to the palm wine, and are also, associated with the aroma, consistency and colour of palm wine by the production of polysaccharides^16,42^. Indeed, one of the broadly recognized advantages of LAB along with acetic acid bacteria (AAB) in food fermentation is the inhibitory effect on foodborne pathogens particularly on the members of Enterobacteriaceae^43^, which could explain the very low relative occurrence (<1%) of the potential pathogenic members of Enterobacteriaceae in the palm wine samples studied, despite the poor hygienic production environment.

Among the Proteobacteria phylum, member of AAB namely *Acetobacter*, *Gluconoacetobacter* and *Gluconobacter* were present in the palm wines. The overall predominance of *Acetobacter* (with an average relative abundance of 21%) in the palm wines of Côte d’Ivoire is unique to this study. At the species level, *Acetobacter pasteurianus* was found prevalent (~18% relative abundance) in the palm wines of Côte d’Ivoire. Indeed, various studies have reported the presence of AAB in the fermented palm sap^44^. *Acetobacter indonesiensis* was reported as the predominant AAB in ‘Bandji’ palm wine of *Borassus akeassii* from Burkina Faso^16^ and *Gluconacetobacter* also identified in the coconut palm wine of Mexico^28^. In addition, the bacterium *Zymomonas*, predominantly present in the Nigerian palm wine^27^ was negligible (~1% relative abundance) in the palm wines of Côte d’Ivoire. Although AAB has been previously identified as wine spoilage bacteria, the population of AAB are often underestimated due to the lack of appropriate cultivation techniques^45,46^. Amplicon sequencing data allowed us to observe a significant population of AAB in the three palm wine types, which also explain the increased susceptibility to spoilage during storage.

Among the diverse yeast species detected in the palm wines samples, *Saccharomyces cerevisiae* dominated this alcoholic fermentation. The importance of *S. cerevisiae* in palm wine fermentation has been reported by various authors^4,5,41,47^. Recently, the population structure analysis demonstrated a palm tree species or palm wine type-specific adaptive evolution of *S. cerevisiae* strains^18^. The other yeast species detected in low abundance, notably *Hanseniaspora valbyensis*, *Hanseniaspora guilliermondii* and *Saccharomycodes* were consistent with previous culture-based reports^16,32^. Whereas, *Saccharomycodes ludwigii* and *Zygosaccharomyces bailii* notably predominated in the palm wine from Cameroon^5^. These non-*Saccharomyces* yeasts with specific flavour-active characteristics can contribute to the unique flavour of the different palm wine types^48^.

It is well known that the nutritional value, flavours and tastes of palm wines are principally related to the chemical composition of various natures, the productions of which naturally come from palm tree sap metabolites, and transformed or produced by the microbial community during sap fermentation. The metabolite profiling by LC-MS analysis showed that palm wine is a chemically rich substrate with a molecular weight ranges from 72 to 714 g/mol, including free sugars, amino acids, organic acids, sugar alcohols, sugar acids, ketones, terpenes, and several unknown compounds with molecular weight at 4 decimal points and the MS/MS spectra not matching with the available databases. Most of the earlier studies reported the volatile components related to the palm wine aroma^49^ and odorants^20^. Enrichment of essential amino acids in palm wine also reported^36^. A recent study on raphia palm wine using LC-MS identified several compounds which were mainly polyphenols and its glycosides, vitamins, and amino acids^13^. Similar to our study, the enrichment of pyrimidine nucleotide sugars in the raphia palm wine also reported^13^. Though our analysis by LC-MS resulted in much higher components, we could not fix the identity of several metabolites due to the limitations in available spectra and possible novel compounds. However, the multivariate analysis clearly differentiated the three palm wine types produced from three palm tree species. When the identity of the differentiating metabolites will be finalised using external standards, this chemical signature could be used as a marker for claiming the uniqueness (flavour or taste) of the different palm wines produced in the Côte d’Ivoire regions^36^. Among the palm wines analysed, oil palm wine enriched with a higher number of metabolites and with the presence of health-promoting compounds like Gevotroline and Mitoxantrone supports its therapeutic potential. Palm wine documented with several therapeutic properties. In addition to already documented gastrointestinal health-promoting properties and risk of cancer reduction abilities^50,51^, raphia palm wine recently reported as a modulator of glucose homeostasis in a rat model of diabetes by enhancing insulin secretion and inhibiting redox imbalance^12^.

Multivariate analyses conducted in the present study revealed a significant difference among the microbial community and metabolite profiles of the three palm wines produced from three different palm tree species. Palm wine is always produced in an open environment and is therefore easily affected by the local environment, resulting in variations in the microorganism community structure and metabolite profiles. Indeed, the differences among these beverages are undoubtedly related to the palm tree species^44^, but also to the destructive tapping process practised in the Côte d’Ivoire region. The oil palm wine tapping is made on the felled palm tree whereas raphia and ron wines on top meristem of live palm trees (Fig. 1).

## Conclusions

The microbial community structure and metabolite profiles of naturally fermented sap from three different palm tree species were investigated using a combined metagenomic and metabolomic approach. This research demonstrates that palm wines produced in Côte d’Ivoire contained a high diversity of bacteria and yeasts, with *Lactobacillus*, *Acetobacter*, *Leuconostoc*, *Fructobacillus*, *Saccharomyces* and *Hanseniapora* as the predominant taxa. The microbial and metabolite profile of Raphia palm wine was distinctly different from oil palm wine and ron palm wine. The high presence of *Leuconostoc* in raphia palm wine appeared as key differentiating taxa among these types of palm wine. Multivariate analysis based on the 765 metabolites profile clearly separated the three palm wine types produced from different palm tree species, among which 21 metabolites differed significantly between the palm wines. Additionally, the metabolically enriched nature of oil palm wine than the other two varieties could be related to the difference in the wine tapping method practised. This chemical signature when finalised could be used as a marker of uniqueness to the palm wines produced in Côte d’Ivoire regions. Most of the differentiating metabolites were present in high concentration in almost all oil palm wine samples. The presence of gevotroline (an antipsychotic agent) and mitoxantrone (an anticancer drug), supporting its therapeutic potential. This work represents a step forward for providing valuable insight into the microbiology of these natural health-promoting beverages and provides a rationale for selecting functional microorganisms with potential biotechnology applications.

## Materials and Methods

### Sample collection

Palm wine samples were collected from July to October 2016 in two different regions of Côte d’Ivoire, chosen based on producers availability, accessibility of production sites and type of palm wine produced. Thus, ron palm wine (*Borassus aethiopum*) was collected in the centre part and oil palm (*Elaeis guineensis*) and raffia palm (*Raphia hookeri*) wines in south Eastern parts of the country. In total, 10 samples of each type of palm wine were collected (in the earlier hours of the morning) directly from producers in sterile containers, stored immediately on ice and transported to the laboratory for analyses. Once at the laboratory, 30 ml of each sample were centrifuged at 12,000 × g. The pellets were washed twice with sterile saline water and frozen at −80 °C for the cultivation-independent microbial community analyses, while the supernatants were used for metabolomics analyses.

### Metagenomic DNA extraction

DNA extraction from palm wine pellets was performed following a modified method described by Keisam *et al*.^52^. Briefly, in a sterile 2 ml screw-cap tube containing 0.5 g of zirconia/silica beads (Biospec Products, USA) and 4 glass beads, the resuspended pellet (0.25 ml) was homogenated with 1 ml of sterile water and then vortexed to 30 s (MoBio vortex) before centrifugation for 10 min at 18000 × g. Four hundred μl of TES buffer [50 mM Tris, 1 mM EDTA, 8.7% sucrose, pH 8] containing 50 KU lysozyme (Sigma-Aldrich, USA), 20 U lyticase (Sigma-Aldrich) and 25 U mutanolysin (Sigma-Aldrich) were added to the pellet and incubated at 37 °C for 1 h. After this step, 25 μl of Proteinase-K (25 mg/mL) (Himedia, India) was added and the mixture was then incubated at 65 °C for 1 h, followed by addition of 300 μl pre-warmed (65 °C) NTS buffer (0.2 M NaCl, 0.1 M Tris, 2% SDS) (Promega, USA) and a new incubation for 10 min at 65 °C. The DNA was extracted by treating once with phenol and twice with chloroform: isoamyl alcohol mixture (24:1) (Merck, India) with centrifugation at 4 °C for 15 min at 15,000 × g. The DNA was precipitated with an equal volume of isopropanol and then dissolved in 50 μl TE buffer (10 mM Tris, 1 mM EDTA) (Sigma-Aldrich). Finally, the extracted DNA was analyzed for DNA quantity and purity (indicated by the absorbance ratio at 260 nm/280 nm) using the NanoDrop spectrophotometer ND-1000 (Thermo Scientific, USA) and stored at −20 °C until further use.

### Barcoded MiSeq amplicon sequencing

The barcoded PCR amplicon library was built separately for bacteria and yeast community analysis. The V3-V4 region of 16S rRNA gene of bacteria was amplified with the forward primer 5′- AYTGGGYDTAAAGNG-3′ and reverse primer 5′- CCGTCAATTCMTTTRAGT-3′^34^ and the fungal ITS2 region was amplified with the forward primer ITS1-F: 5′-CTTGGTCATTTAGAGGAAGTAA-3′ and reverse primer ITS2: 5′-GCTGCGTTCTTCATCGATGC-3′^53,54^. The primer sequences (IDT, USA) were modified by the addition of a multiplex identifier sequence (barcode) of 8 bp for bacteria^34^ and 12 bp for yeast^55^, which allows the pooling of amplicons. PCR reaction mixture (50 μl) contained 100 ng of template DNA, 1X high-fidelity reaction buffer, 1.0 mM MgCl_2_, 0.1 μM each primer, 0.5 U Phusion high-fidelity DNA polymerase (New England Biolab, USA) and nuclease-free water. The PCR program (C1000 Touch Thermal Cycler, Biorad) was carried out under the following conditions: 98 °C for 5 min; 28 cycles of 98 °C for 150 s, 55 °C (for bacteria)/52 °C (for yeast) for 30 s and 72 °C for 30 s; and 72 °C for 5 min. A template-free reaction was maintained as a control. The amplified PCR products were verified in a 2.0% agarose gel (w/v) (Promega,USA) and purified using QIAquick gel extraction kit (Qiagen, New Delhi, India). Further, quantified using Qubit dsDNA BR Assay Kit (Invitrogen) in a Qubit 2.0 fluorometer (Invitrogen, Carlsbad, CA). The amplicon samples were pooled in equimolar proportions. Finally, the multiplexed amplicon pool for bacteria and yeast were mixed in an equimolar proportion. The final amplicon pool was sent to the NGS facility in Xcelris Genomics (Ahmedabad, India) for adapter-ligated paired-end Illumina MiSeq sequencing reaction.

### Nucleotide sequence data processing

The raw sequence reads obtained was processed using the QIIME v1.8.0 bioinformatics pipeline^56^. Briefly, removal of adaptor sequences, generation of paired-end reads, sample demultiplexing^34^ and the segregation of yeast and bacterial sequences was performed using in-house Perl scripts. The segregated yeast and bacterial sequences were separately clustered into operational taxonomic units (OTUs) at 97% identity threshold by using the furthest-neighbour algorithm. For yeast community analysis, the representative OTU sequences were taxonomically annotated using the UNITE fungal ITS database release version 7.1 (http://unite.ut.ee). For bacterial taxonomic annotation, MG-RAST pipeline was used at 97% similarity threshold against the M5RNA database.

### Metabolites profiling by LC-HRMS

The Dionex Ultimate 3000 ultrahigh performance Liquid chromatography (UHPLC) coupled to a Q-Exactive Orbitrap (Thermo Fisher Scientific) of C-CAMP MS Facility, Bengaluru was used for the metabolite profiling of palm wine by following the method of Padma *et al*.^57^.

For the sample preparation, one ml of the palm wine sample supernatant was diluted in 1 ml of acetonitrile (1:1 dilution). The mixture was centrifuged at 18,000 × g for 10 min at 4 °C and filtered through 0.2 µm PTFE filter into a 2 mL septum vial and stored at −80 °C for LC-MS analysis. Before analysis, all the samples were thawed in ice and vortexed well. Taurocholate was used as an internal standard for quality control. The chromatography was performed on a hydrophilic interaction liquid chromatography column (HILIC, 5 μ, 150 mm × 4.6 mm, Phenomenex Luna) with a flow rate of 0.4 mL/min and maintained at 40 °C. The mobile phase-A contained 5 mM ammonium acetate in water and the phase-B contained 5 mM ammonium acetate in water with acetonitrile in a ratio of 1:9. The run gradient was 100% B to 0% B over a time period of 45 minutes and regained to 100% B at 46 min, and maintained at 100% B up to 55 min. The Q-Exactive Orbitrap instrument was set up for data acquisition in the full scan/data-dependent scan (FS/DDS) mode in a mass range of 70 to 750 m/z, alternating between MS and MS/MS scans. The full scan was set from 70000 to 140000 resolution. The run was performed in the negative ionization mode with a spray voltage of 2500 V, 320 °C vaporizer temperature, sheath gas flow rate of 40 arbitrary units, and auxiliary gas flow rate of 10 arbitrary units. The spiked taurocholate was used as an internal standard and later used for intensity normalization of the data. The raw data files were imported into SIEVE 2.2 for the generation of peak list and component extraction, and the Human Metabolome DataBase/Kyoto Encyclopedia of Genes and Genomes (HMDB/KEGG) were used for possible identification of the compounds. From the pooled quality control samples data, the coefficient of variation (CV) was calculated and the data with CV_QC > 20% were removed^58^.

### Statistical analysis

The relative abundance data of the bacterial and yeast OTUs at different taxonomic levels were used for performing statistical analysis. The yeast and bacterial data were combined together for analyzing the overall microbial community structure difference between the palm wine samples collected from three different types of palm tree species. Principal coordinate (PCoA) analysis was performed with the Bray-Curtis dissimilarity and the significance of the difference between the palm wine types was tested by PERMANOVA test with 10,000 permutations using Bray-Curtis distances in PAST v3.22^59^. The Wilcoxon test was conducted to show the significance of the difference in the abundance of taxa between the paired groups by using “svDialogs” in R package (v3.5.2) and expressed as “Benjamini-Hochberg” (BH) corrected p-value. To calculate the diversity indices (Chao species richness and Shannon diversity)^60^, the species-level OTUs of yeast and bacterial data together was used in PAST v3.22 and the boxplots were visualised using BoxPlotR (http://shiny.chemgrid.org/boxplotr/) to show the significant differences (Students t-test, 2 tailed). Similarly, the palm wine metabolite data were log-transformed (log x_i +_ 1) and analysed by PCoA using Bray-Curtis dissimilarity. The hierarchically clustered heat map was visualized using “gplots” in R for the microbial species-level OTUs and palm wine metabolites that were significantly different (log2 fold change of >1 and p < 0.0001)^61^ between the three palm wine types with a colour key representing the intensity of the value.

## Data Availability

The yeast ITS sequences generated in the present study was deposited in NCBI SRA with the accession numbers PRJNA507709 and the bacterial 16S rRNA sequence datasets supporting the conclusions of this article are available in MG-RAST (https://www.mg-rast.org/mgmain.html?mgpage=project&project=mgp83825).
